# The entropy of chaotic transitions of EEG phase growth in bipolar disorder with lithium carbonate

**DOI:** 10.1038/s41598-021-91350-9

**Published:** 2021-06-04

**Authors:** Rüştü Murat Demirer, Sermin Kesebir

**Affiliations:** 1 Üsküdar University, İstanbul, Turkey; 2 Üsküdar University, NPİstanbul Brain Hospital, Ahmet Tevfik İleri C. N: 18 Ümraniye, İstanbul, Turkey

**Keywords:** Neuroscience, Physiology, Psychology, Biomarkers, Medical research, Mathematics and computing

## Abstract

The application of chaos measures the association of EEG signals which allows for differentiating pre and post-medicated epochs for bipolar patients. We propose a new approach on chaos necessary for proof of EEG metastability. Shannon entropies of concealed patterns of Schwarzian derivatives from absolute instantaneous frequency transformations of EEG signals after Hilbert transform are compared and found significantly statistically different between pre and post-medication periods when fitted to von Bertalanffy’s functions. Schwarzian dynamics measures was compared at first baseline and then at the end of the first hour of one dose 300 mg lithium carbonate intake for the same subject in depressive patients. With an application of Schwarzian derivative on the prediction of von Bertalanffy’s models, integration and segregation of phase growth orbits of neural oscillations can be understood as an influence of chaos on the mixing of frequencies. A phase growth constant parameter was performed to determine the bifurcation parameter of von Bertalanffy’s model at each given non-overlapped EEG segment. Schwarzian derivative was sometimes very close positive near the origin but stayed negative for most of the number of segments. Lithium carbonate changed the chaotic invariants of the EEG Schwarzian dynamics and removed sharp boundaries in the bipolar spectrum.

## Introduction

Bipolar disorder is characterized by recurrent depressive and manic episodes^1^. A mixed episode offers possibility to evaluate compensatory regulation effort with affective dysregulation. The brain is a complex adaptive system that actively minimizes the entropy through our sensory and physical states. The quasi-attractor states are producing self-organized action-perception cycles from higher cortical states to lower cortical states vice versa^2^. There exists a mathematical field theory of the hierarchically organized emergence dynamics (i.e. creation and annihilation) of spatial and temporal dimensions at micro, meso, and macro scales by behavioral patterns in mental processes. Haken introduced a circular causality (both top-down and bottom-up approach), order parameter and complexity reduction in relation to those processes. Typical cortical transitions are not merely random but are in the framework of transitory and chaotic dynamics with fractal or nested modular hierarchy^3,4^. The chaotic itinerancy among quasi-attractors are defined as memories which allow the brain to learn new input patterns while maintaining memories in the situation of human communication. Memory is built in hierarchical architecture as simple memory, episodic memory and relational memory. Chaotic itinerancy appears in bifurcating processes at different levels of brain regions. This stage of higher memory formation can be a marker through communication as output-driven information processing^5^.

**Figure 1:**
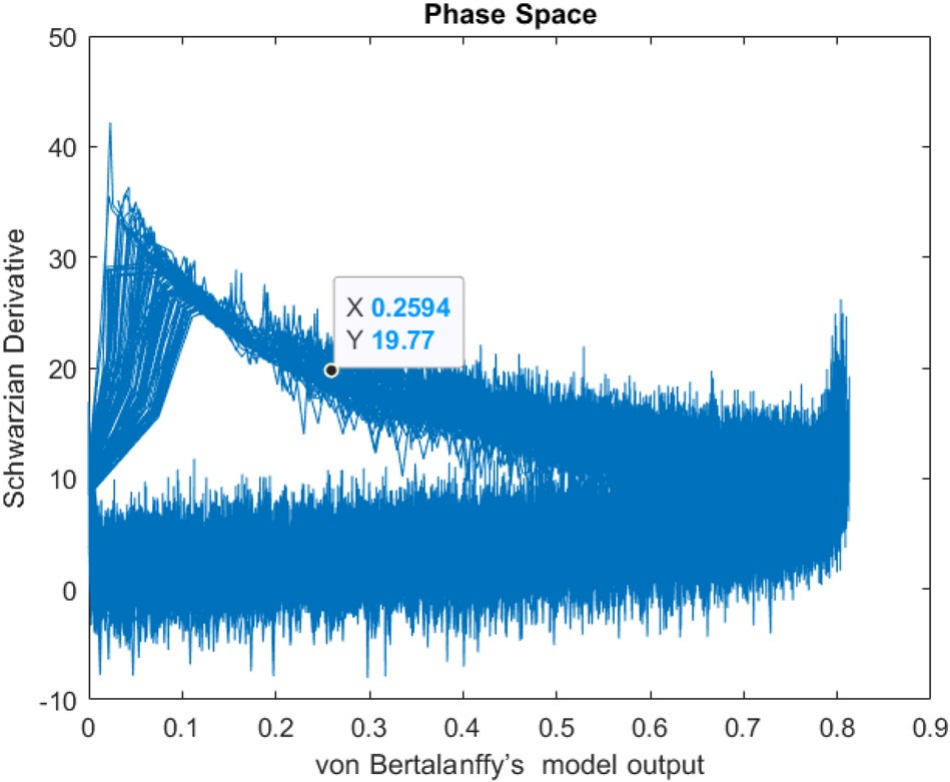
Phase space of model output with the logarithmic Schwarzian derivative (attractor).

Collective dynamics of neural populations oscillate across a wide range of topologically transitive states. These are aperiodic, unbalanced, transitive and density oscillations in different brain regions that live in a high-dimensional electrode space of whole cortical neuronal activity^6–8^. The EEG oscillations cause the appearance of a temporal sequence of aperiodic quasi-attractors which are weakly spatially coupled together for cognitive processes.The previous studies were limited to low dimensional EEG amplitude dynamics. Our approach is based on the transitory dynamics which is typically observed in both spatial and temporal resolution of the phase of oscillations in 10–20 channel scalp EEG. We improved on chaotic itinerancy, describing the trajectories of quasi-attractors as shown in Fig. 1 and the transition rules of EEG phase dynamics by adjusting the single individual growth rate and asymptotic parameters of the model assigned to each channel and segment individually^3^.

**Figure 2:**
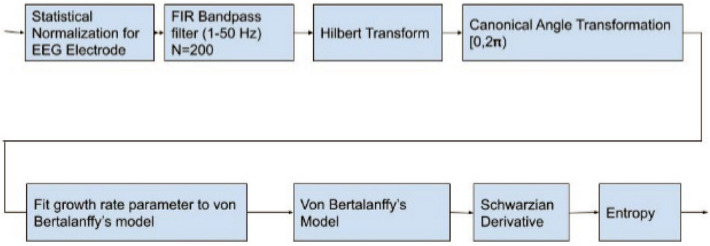
The general architecture of chaotic model for each electrode associated with Schwarzian derivative.

We are based on the collective dynamics of neural populations. With an application of the Schwarzian derivative of von Bertalanffy’s models, integration and segregation of phases of EEG oscillations spread through different regions can be explained as a causality of chaos. We proposed a new method that captures concealed chaotic patterns and transitive points of rhythms as shown in Fig. 2. The Schwarzian derivative value shows a good continuity and monotonicity of topological entropy in a wide range of oscillations and depicts the order in the succession of transitive bifurcations in cognitive processes^6–10^. The Schwarzian derivative can explain the flow of phase/frequency transitions in the EEG band. In our approach, the Schwarzian derivative was negative in our one-dimensional unimodal model function creating a convexity for a given growth parameter estimation due to von Bertalanffy’s conditions satisfied throughout the complete interval of the dynamic variable with chaotic behaviour^10–15^. This was helping us to localize at most one stable periodic attractor. In some growth parameter estimation values, we allowed the unimodal functions to get a negative Schwarzian derivative value which was transformed to convex maps in some subsets $x \in S^{ - }$ of the time interval (segment). However, the Schwarzian derivative is sometimes positive in a neighborhood of the fixed point $x = 0$ which will cause non-convexity of the unimodal function which determines positive Schwarzian denoted sequences of (coexisting) attractors of different periods in some subsets $x \in 0^{ + }$ due to singularities. Schwarzian derivative may be positive in the initial few percent of the parameter space. Periodic attracting orbits $O_{m}$ and $O_{n}$ of integer periods $m$ and $n$ will be observed like period-tripling in this case^4^.

We can differentiate pre and post-medicated epochs of Schwarzian dynamics for bipolar patients. Negative Schwarzian derivatives of phase guarantee positive entropies, continuity, and monotonicity of topological entropy. We are then able to understand how the topological entropy depends on chaotic itineraries within each segment. We found significantly statistically differences in the topological entropies in terms of many stable orbits and the succession of chaotic itineraries depending on intrinsic growth rate $r$ for each segment between pre and post-medication periods. The progress of unstable periodic orbit, toward a stable state, could be quantified with Shannon entropy in Schwarzian dynamics. Schwarzian dynamics also gave us the existence of an upper limit to the number of stable orbits and the non-existence of wandering intervals of those concealed growth patterns of instantaneous frequency valuations.

In conclusion, this study aimed to investigate chaotic phase transitions in the bipolar spectrum. For this purpose, the entropy of chaotic transitions was compared at baseline and the first hour of 300 mg lithium carbonate intake.

## Methods

### Sample

In our study, patients with major depressive episode, recurrence, current episode moderate or severe were evaluated consecutively according to DSM-V (n = 10). Our inclusion criteria were.

1. Psychotic findings,
2. Atypical features and/or mixed symptoms,
3. Presence of bipolar disorder in family history,
4. Cyclothymic or hyperthymic temperament.

The presence of comorbidity and chronic physical illness exclusion criteria for any psychiatric disorder. In addition, patients were not required to take medication for current depression.

### Assessment

The permission required for this study was obtained from the ethics committee of our university. After each diagnostic interview, HDRS (Hamilton Depression Rating Scale), MDQ (Mood Disorder Questionnaire) and TEMPS-A (Temperament Evaluation of Memphis, Pisa, Paris and San Diego Autoquestionnaire) were applied. EEG was performed. 300 mg of lithium carbonate was then administered orally. EEG was repeated at the end of the first hour.

EEG was recorded at a sample rate of 125 samples/s. Impedances for each electrode referring channels were kept below 30 kΩ. EEG processed offline for artifact rejection. A high pass filter was applied at 0.1 Hz and a low pass filter was applied at 70 Hz.

### Statistical analysis

Comparisons were made with SPSS 20.0 Wilcoxon test before and after medication.

### Mathematical approach of chaotic EEG brain dynamics embedded in phase growth models

The general system architecture of system architecture for each electrode is shown in Fig. 2. The analytic phase $\varphi \left( t \right)$, in radians is derived by using the Hilbert transform and transformed to canonical angle in MATLAB. The EEG channel, for each $j.$ electrode ($j = 1, \ldots 21$), is denoted by $v_{j}$ on each segment. $v_{j}^{\prime }$ is from the Hilbert transform of $v_{j}$.

1 $$ u_{j} \left( t \right) = v_{j} \left( t \right) + iv_{j}^{\prime } \left( t \right) $$

where $u \in {\mathbb{C}}$ is the *analytic signal.* The Hilbert transform is well known and is expressed as

2 $$ v_{j}^{\prime } \left( t \right) = p \cdot v \cdot \frac{1}{\pi }\mathop \int \nolimits_{ - \infty }^{\infty } \frac{{v_{j} \left( {t^{\prime } } \right)}}{{t - t^{\prime } }}dt^{\prime } = \mathop {lim}\limits_{\epsilon \to 0} \frac{1}{\pi }\mathop \int \nolimits_{{\left| {t - t^{\prime } } \right| > \epsilon }}^{{}} \frac{{v_{j} \left( t \right)}}{{t - t^{\prime } }}dt^{\prime } $$

for $t = 1, \ldots N$ indices for each given segment at a given channel, $j$. Hilbert transform provides a real and imaginary part of embedding an inherent strange attractor. $v_{j}^{\prime } \left( t \right)$ denotes that a Hilbert transform of the original signal has been performed. $dt$ is a Lebesgue measure corresponding to the reciprocal of the sampling frequency. The analytic signal, $u_{j} \left( t \right):{\mathbb{R}} \to {\mathbb{C}}$ is obtained by combining $v_{j} \left( t \right)$ and $v_{j}^{\prime } \left( t \right)$. We can obtain an instantaneous phase that shows the EEG oscillatory behavior how evolves in time. At each sampling step, we obtain one point in a complex plane. The analytic instantaneous amplitude of each point can be represented by

3 $$ A_{j} \left( t \right) = \left[ {v_{j}^{2} \left( t \right) + v_{j}^{\prime 2} \left( t \right)} \right]^{0.5} $$

and the arctangent of each sample point will give us the instantaneous analytic phase value at a $t$,

4 $$ \varphi_{j} \left( t \right) = atan\left[ {\frac{{v_{j}^{\prime } \left( t \right)}}{{v_{j} \left( t \right)}}} \right] $$

for $j = 1..21$ EEG channels. The instantaneous frequency of the signal is defined as

5 $$ \varphi^{\prime }_{j} \left( t \right) = \frac{d\varphi \left( t \right)}{{dt}} $$

the analytic phase was discontinuous because each time $v\left( t \right)$ went to zero, the tangent went to infinity, and the analytic phase jumped from $+ \pi /2$ to $- \pi /2$. The phase values $\varphi_{j} \left( t \right)$ are unwrapped, differentiated and finally getting absolute values as the radian phase angles which are transformed from $\left[ { - \pi ,\pi } \right]$ to positive $\left[ {0, 2\pi } \right)$ range. We define this transformation as $\varphi_{j}^{\prime G} \left( t \right)$ for each time segment then substitute the values into the von Bertalanffy model as an instantaneous frequency series. In each electrode, dynamic representations of data are segmented into fixed time intervals in a non-overlapped manner that uses inter variability. We then consider one dimensional EEG dynamical model independently for each channel represented by von Bertalanffy’s function of instantaneous frequency values $f_{r} \left( x \right):\left[ {0,1} \right] \to \left[ {0,1} \right]$. We omitted the channel index, $j$ for the sake of simplicity. Then the model

6 $$ f_{r} \left( x \right) = rx^{2/3} \left( {1 - x^{1/3} } \right) $$

with $x = \frac{{\varphi_{j}^{\prime G} \left( t \right)}}{{\varphi_{\infty }^{^{\prime}} }} \in \left[ {0,1} \right]$ the normalized phase of canonical instantaneous frequency values, $t$ is the discrete-time index defined in each time segment. The minimum frequency value on each segment is subtracted from all values in each segment causing zero growth change initially to start from zero. This is a correction factor for each segment. Intrinsic growth rate depends on two-parameter spaces determined by each segment in neural state space.

7 $$ r = r\left( {K,\varphi_{\infty }^{\prime } } \right) = \frac{K}{3}\varphi_{\infty }^{\prime 2/3} > 0 $$

is an intrinsic growth rate of the EEG phase for each segment at a given electrod. $\varphi_{\infty }^{\prime }$ is the asymptotic local frequency value. The intrinsic growth rate parameter $r$ of von Bertalanffy’s model which depends on a spectral measure, $K$ influences the associated Lyapunov exponent.

Given any interval (each time segment) $I \subset {\mathbb{R}}$ and $x \in L^{1} \left( I \right),$ we define Bertalanffy growth rate constant, $K$ defined as a spectral measure which is finite logarithmic integral for each segment. Integral over instantenous phase vales denote phase growth dynamics.

8 $$ K = \left\langle f \right\rangle_{I} = - \frac{1}{\left| I \right|}log_{2} \left( {\int_{I}^{{}} {x dx} } \right) .$$

The model of phase growth dynamics for each channel (electrode) satisfies the following conditions:

- $f_{r}$ is continuous on [0, 1]
- $f_{r}$ has a unique critical phase value $c = \left( \frac{2}{3} \right)^{3} \in \left( {0,1} \right)$ of normalized $x$ value.
- First-order derivative $f_{r}^{\prime } \ne 0,\forall x \in \left( {0,1} \right)\backslash \left\{ c \right\}, f_{r}^{\prime } \left( c \right) = 0$ and second-order derivative, $f_{r}^{\prime \prime } \left( c \right) < 0$
- The model dynamics $f_{r} \in {\text{C}}^{3}$ must be three times differentiable.

Finally, we can explore the chaotic dynamics of the phase growth model by analyzing the Schwarzian derivative given by

9 $$ {\mathcal{S}}\left( {f_{r} \left( x \right)} \right) = \frac{{f_{r}^{\prime \prime \prime } \left( x \right)}}{{f_{r}^{^{\prime}} \left( x \right)}} - \frac{3}{2}\left( {\frac{{f_{r}^{^{\prime\prime}} \left( x \right)}}{{f_{r}^{^{\prime}} \left( x \right)}}} \right) $$

${\mathcal{S}}\left( {f_{r} \left( x \right)} \right)$ shows the bifurcation stability of the EEG phase dynamics. A pre-Schwarzian derivative is defined as

10 $$ T_{f} \left( z \right) = \frac{{f_{r}^{^{\prime\prime}} \left( x \right)}}{{f_{r}^{^{\prime}} \left( x \right)}} $$

which explains deviation from linearity. Schwarzian is a linear operator whereas Pre-Schwarzian derivative is a nonlinear operator.

In terms of flip bifurcations, ${\mathcal{S}}\left( {f_{r} \left( x \right)} \right)$, we define the transitions between the stability dynamics and the period-doubling dynamics at a beginning value of $r = \frac{5}{3}$. This $r$ value is the chaotic semistability i.e. metastability dynamics which corresponds to the transitions among chaotic conditions and depicts no continuous stable admissible condition. In other words, brain dynamics has a distinct attractor jumping intermittently from one to another which can disappear in a small range to cause a new attractor. In the new attractor, EEG trajectories of brain dynamics would stay for a longer time in different regions of the brain^9^ as shown in Fig. 1. Then, the entropy of phase transition dynamics for each electrode can be defined as Shannon entropy for each segment and channel.

11 $$ \Delta E: = - \mathop \sum \nolimits_{{{\mathcal{S}}\left( {f_{r} \left( x \right)} \right) \in {\mathcal{H}}}} p_{{{\mathcal{S}}\left( {f_{r} \left( x \right)} \right) }} logp_{{{\mathcal{S}}\left( {f_{r} \left( x \right)} \right) }} $$

where the probability ${\text{p}}_{{{\mathcal{S}}\left( {f_{r} \left( x \right)} \right)}} : = \wp \left( {{\upxi } = {\mathcal{S}}\left( {f_{r} \left( x \right)} \right)} \right),{\mathcal{S}}\left( {f_{r} \left( x \right)} \right) \in {\mathcal{H}}$. A random variable ${\upxi }$ takes values in a finite set, ${\mathcal{H}}$ domain of Schwarzian values which take mostly negative values. Shannon entropy is a quantification of measure on correlations among the successive values of the phase changes involved in highly chaotic, almost ergodic, single-dimensional maps.

### Ethics approval and consent to participate publication

The Institutional Review Board of Uskudar University approved the study. QEEG is a routine evaluation tools in our NPİstanbul Brain Hospital outpatient clinic. Patients gave written informed consent in accordance with the Declaration of Helsinki.

### Consent for publication

Patients gave written informed consent for publication.

## Results

The mean age of the patients was 43.6 ± 5.7 years and the mean age of onset was 20.3 ± 2.8 years. The mean number of depressive episodes was 3.5 ± 1.2. HDRS score was 27.4 ± 3.7, MDQ score was 13.4 ± 1.1, cyclothymic and hyperthymic temperament scores were 17.8 ± 1.2 and 21.3 ± 2.1.

Comparisons were made with Wilcoxon test before and after medication which is shown in Table 1. Classification performance represents based on all electrodes. Entropy change (∆E) can be expanded on single electrodes which can potentially provide doctors a clinical biomarker.

**Table 1:** p-associations to Wilcoxon-sign test of entropy values of Schwarzian chaos between pre- and postmedication over 10 bipolar patients for 21 electrodes each over 30,000 time points at a 125 Hz sampling frequency corresponding to 4 min. recording periods.

Patient no	*P* value
1	0.0595697790745 e−03
2	0.0689875106961 e−03
3	0.0595697790745 e−03
4	0.10641560703 e−03
5	0.059569779074 e−03
6	0.059569779074 e−03
7	0.059569779074564 e−03
8	0.079802219389292 e−03
9	0.079802219389292 e−03
10	0.059569779074564 e−03

**Figure 3:**
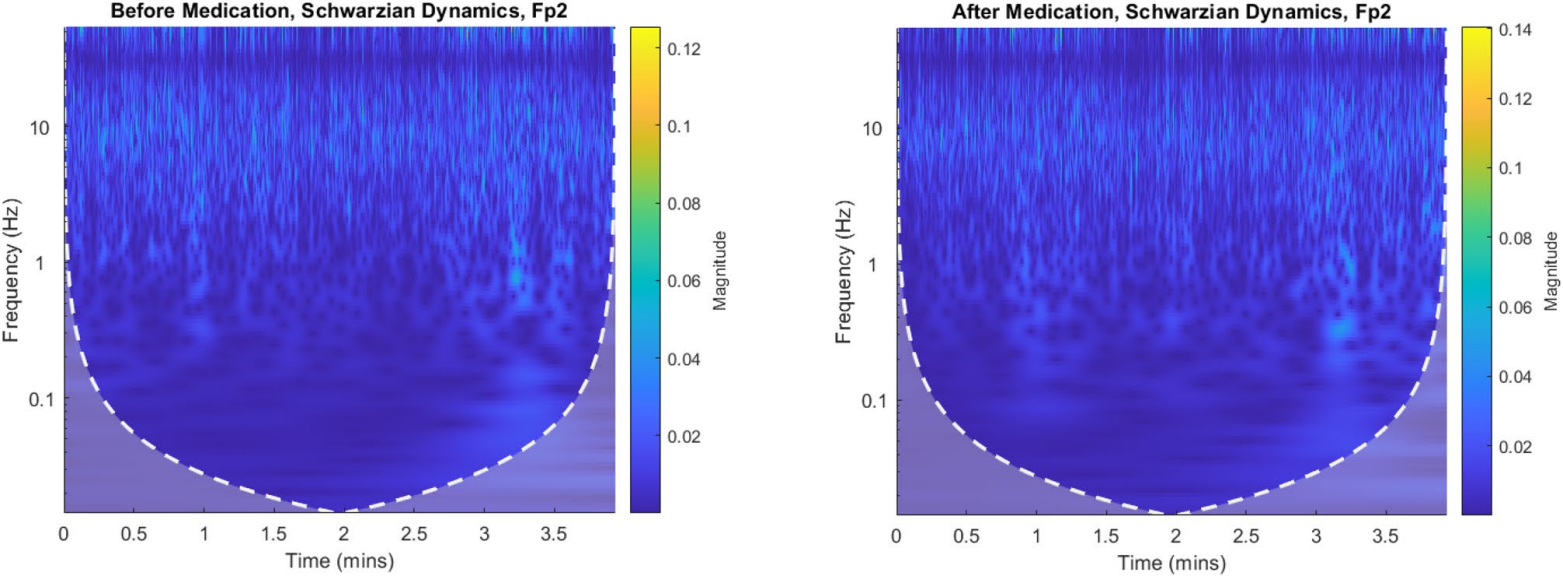
Continuous wavelet transformation of Schwarzian Dynamics (pre-intake and post-intake).Instantaneous frequency transitions in vertical direction are seen clearly.

We founded that EEG dynamics covers a wide range of a scrambled set of frequencies through the periodic doubling of across delta, beta, alpha, gamma, etc. bands in a given topology as shown in continuous wavelet transform of Schwarzian dynamics (Fig. 3).

**Figure 4:**
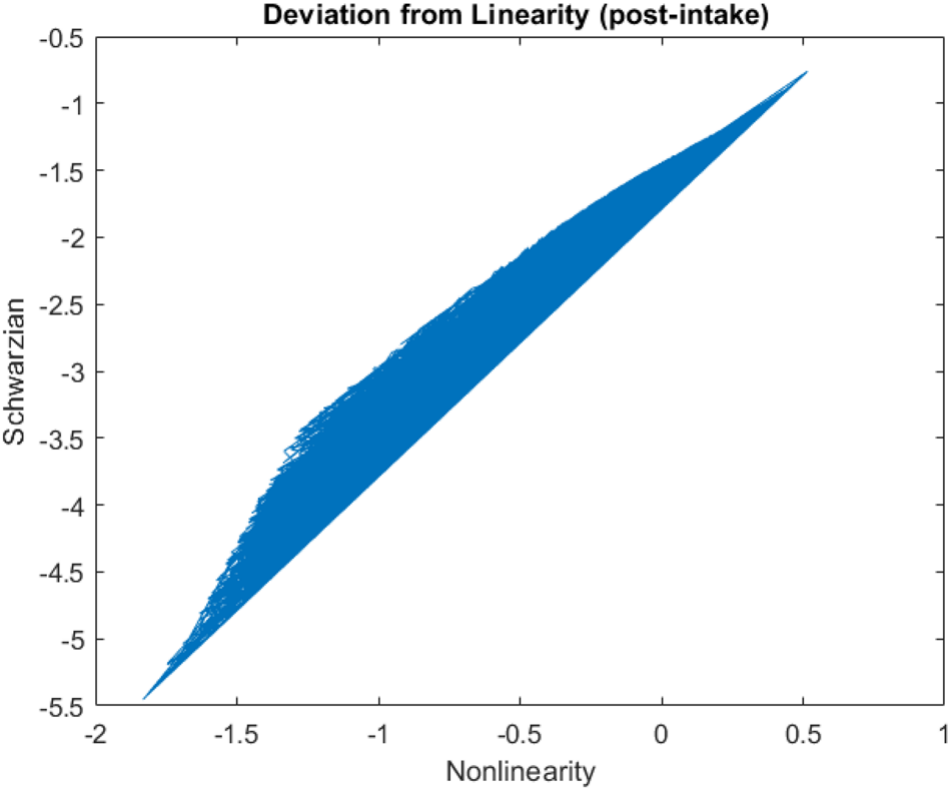
Shwarzian derivative of the model versus pre-Schwarzian derivative, ${\text{T}}_{{\text{f}}} \left( {\text{z}} \right)$ as a non-linearity post-intake term (the slope of the linearity (gain) is higher and Schwarzian negativity is stronger).

**Figure 5:**
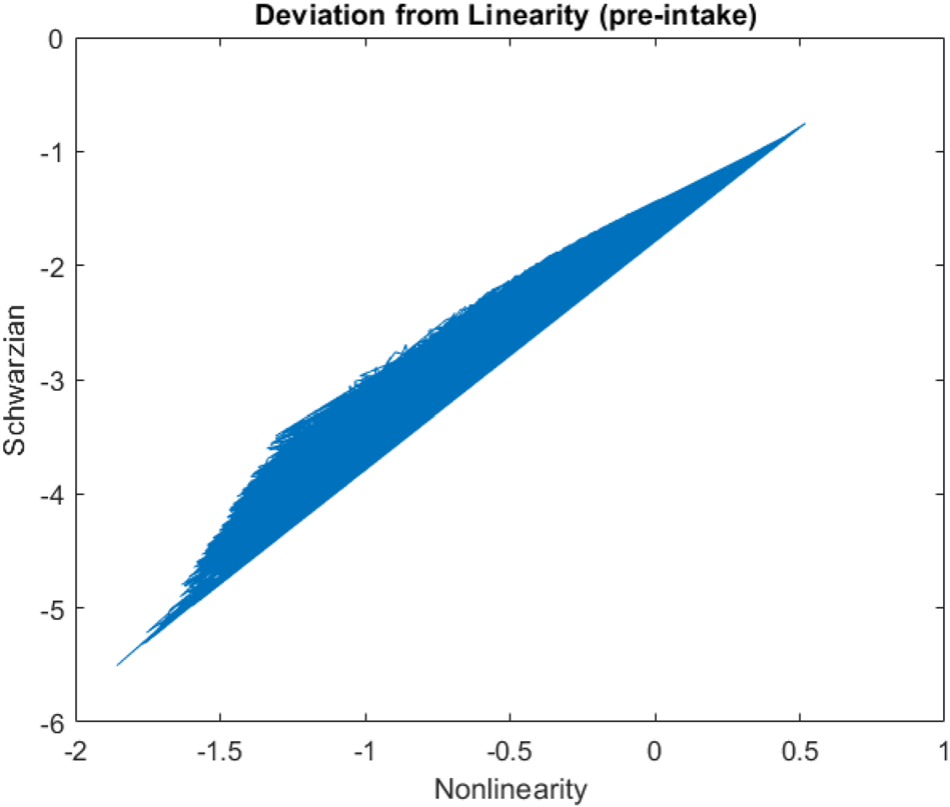
Shwarzian derivative of the model versus pre-Schwarzian derivative: pre-intake term.

Figure 4 and Fig. 5 shows deviation of the phase from the linear growth for both pre-medication and post-medication patients which is Pre-Schwarzian derivative. The linear growth slope is different and higher after post-medication. Besides the nonlinearity region is more than the pre-medication period.

**Figure 6:**
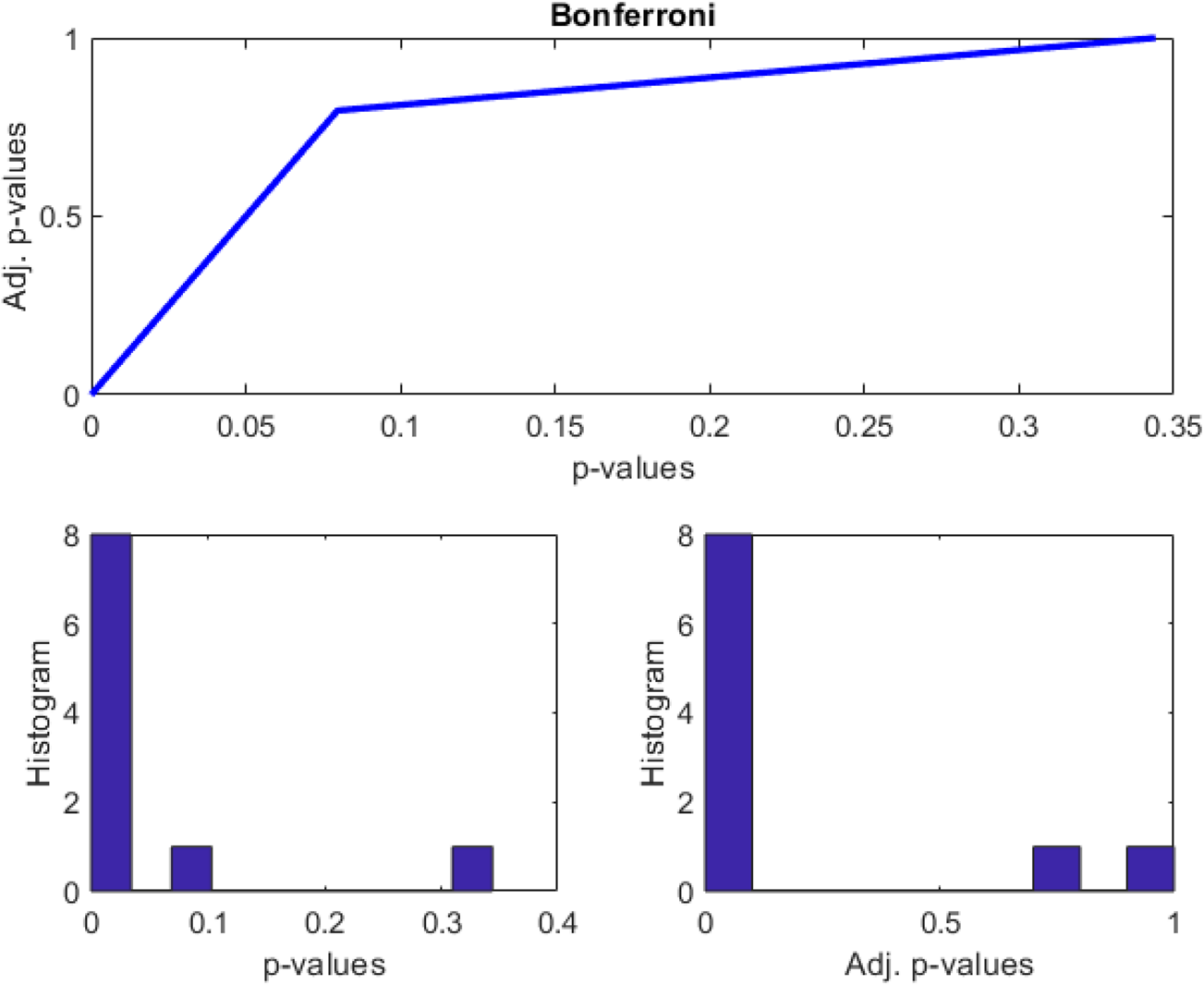
fwer_holmbonf: Holm-Bonferroni correction of the FWER (also known as sequential Bonferroni).

We used a two-sample t-test on the entropy distributions of Schwarzian dynamics of von Bertalanffy’s models from all channels (electrodes) at the confidence level $\alpha = 0.001$ as shown in Tables 2 and 3. Holm-Bonferroni corrections of the FWER from p-values of t-tests are $\le 0.001$ for eight patients among a total of 10 patients as shown in Fig. 4. Bonferroni corrected *p* values, the adjusted significance level, the number of rejected null hypotheses are performed after the t-test is applied as shown in Fig. 6^15^.

**Table 2:** Test statisitics of pre-intake and post-intake comparison.

1	2	3	4	5	6	7	8	9	10
36.170	8.5373	9.5402	6.7794	22.492	12.887	7.2275	6.5110	7.9458	6.4282

**Table 3:** Confidence interval for the difference in entropy means of pre-intake and post-intake comparison, returned as a two-element vector containing the lower and upper boundaries of the 100 × (1 – Alpha)% confidence interval.

1	2	3	4	5	6	7	8	9	10
0.282	0.041	0.049	0.0196	0.217	0.0567	0.0241	0.0658	0.0686	0.1129
0.232	0.101	0.109	0.0634	0.299	0.0999	0.0710	0.0192	0.0261	0.0324

**Figure 7:**
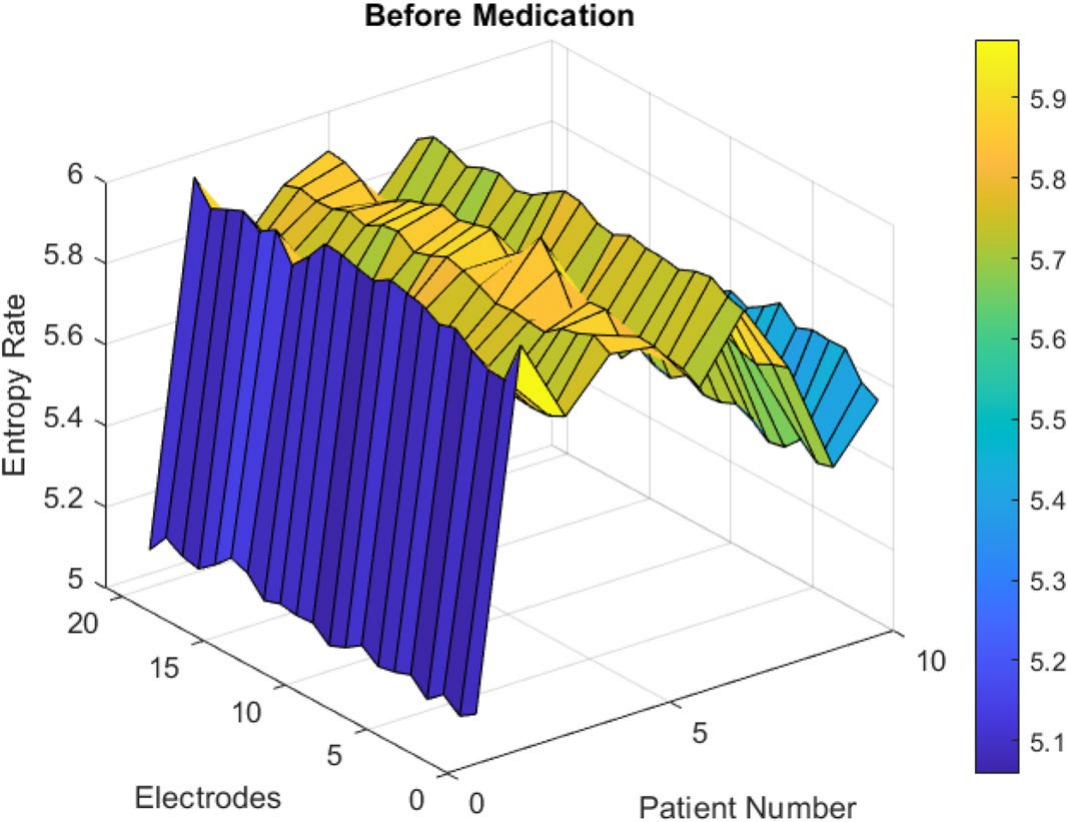
Before medication, Entropy Rate (∆E) is high in bipolar patients, disordered over electrodes.

**Figure 8:**
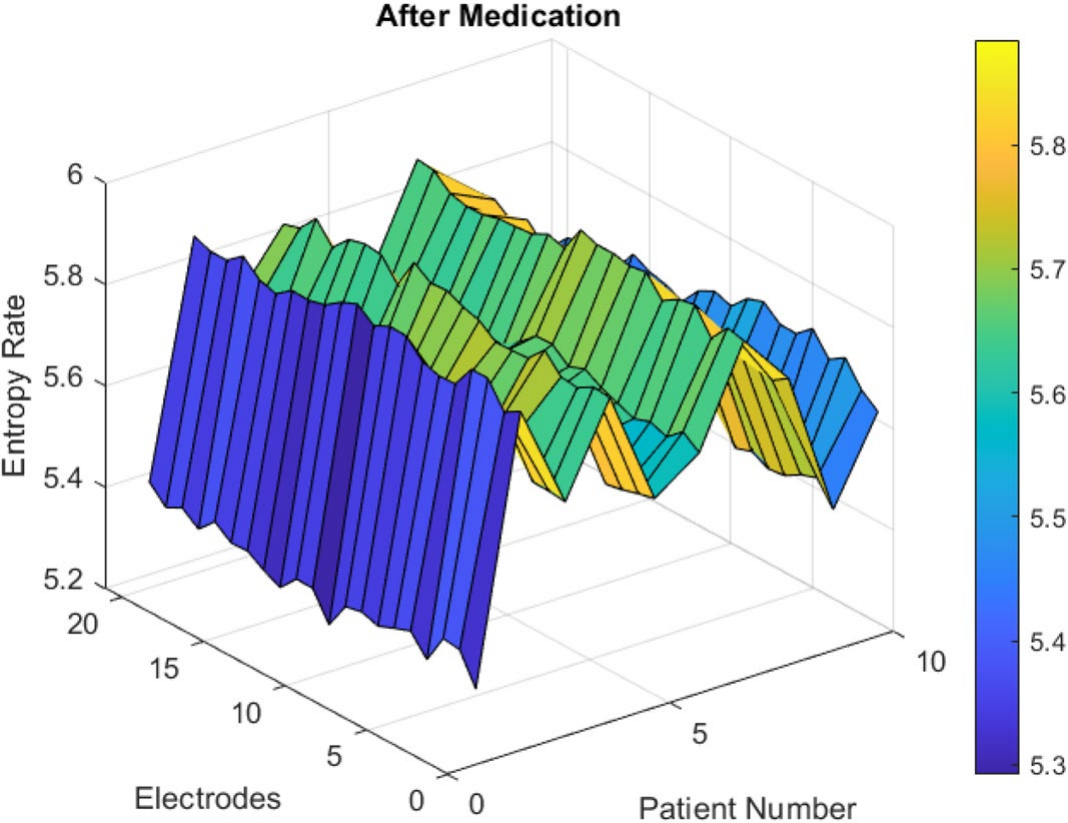
Post- intake, (∆E) Entropy Rate is low in bipolar patients, disordered over electrodes.

Besides, the *p* values of entropies for 10 patients based on 20 electrodes are given in Table 1 for Wilcoxon signed-rank test from pre and post-medication terms on all electrodes. $p$-associations to Wilcoxon-sign test of entropy were agreed on less than $\le 0.001$ for all patients. This approach claimed a significant entropy change of Schwarzian dynamics in the whole-brain network that is specific to Lithium carbonate medication as shown in Figs. 7 and 8.

**Figure 9:**
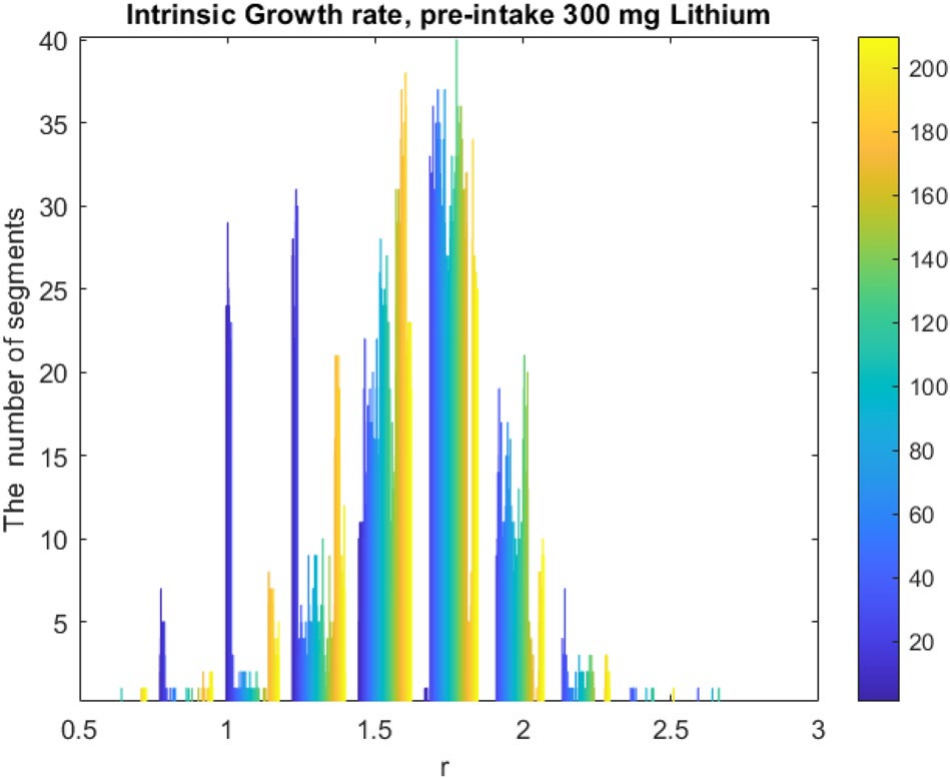
Intrinsic Growth rates for 10 patients each 20 channel and 59-time segments for pre-intake (6018-period doubling chaotic regions).

**Figure 10:**
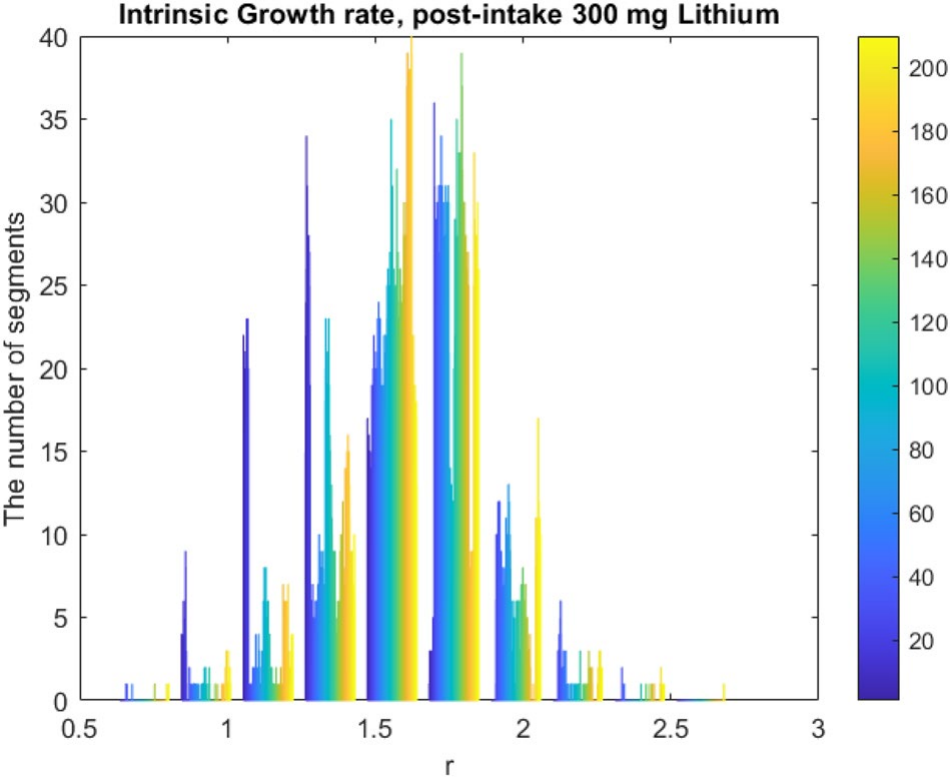
Intrinsic Growth rates for 10 patients each 20 channel and 59-time segments for post-intake (5239-period doubling chaotic regions exist).

The model explains period-doubling and chaotic itinerancy if $\frac{5}{3} < r < \frac{{3^{3} }}{{2^{2} }},$ of the phase growth dynamics which means that the interval $\left[ {f_{r}^{2} \left( c \right),f_{r} \left( c \right)} \right]$ is forward invariant with the basin of attraction $\left] {0,1} \right[$ for each segment.The number of quasi-attactors as intrinsic growth rates are labeled as shown in Figs. 9 and 10. This invariant is the succession of the presence of unstable fixed points in phase space which causes a scrambling effect over 1–50 Hz. through a wide range of frequencies that might explain cognition in healthy subjects and mental disorders.

We obtained the plots using MATLAB and Statistics and Signal Processing Toolbox Release 2020b, The MathWorks, Inc., Natick, Massachusetts, United States^16^.

## Discussion

**Figure 11:**
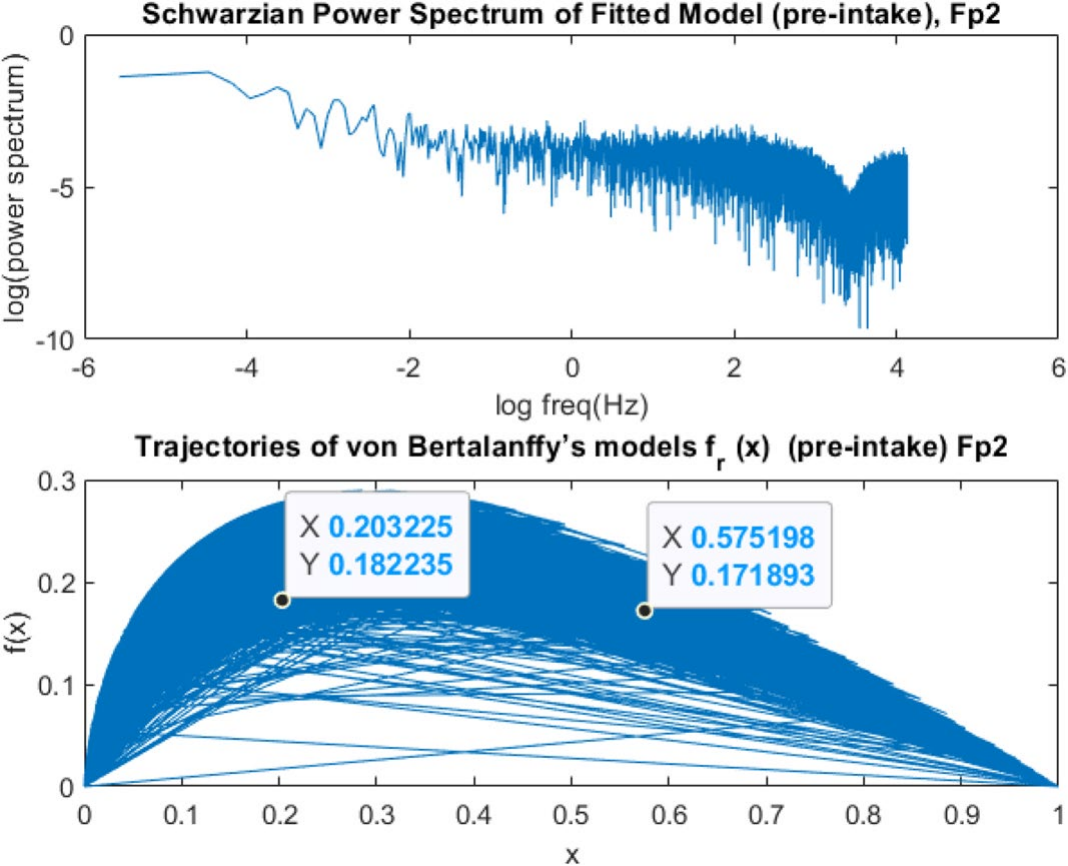
Power Spectrogram of Schwarzian coupling and von Bertalanffy’s EEG Phase Growth Model before medication for Fp2 electrode for an interval of 30,000-time points of T = 500 time points for each segment at fs = 125 Hz.

Chaotic itinerancy appears in the brain activity of communicating with people in the form of chaotic phase transition disorders between synchronization and desynchronization^6^. The brain resists a tendency to this disorder and therefore minimizes the entropy change of their sensory states as shown in Fig. 11. Measures for energy, entropy, and temperature can be defined with models using the Carnot cycle and then using the Rankine cycle to incorporate criticality and phase transitions^7^. The knowledge is expressed with the dynamics of two interactive fields of neural activity, one at high and the other at low energy density, and the two operators that create and annihilate the fields. The extremely high density of brief energy in cortical activity patterns can account for the vividness, richness of associations, and emotional intensity of memories in widespread task conditions^8^.

Successful interregional communication depends on the transient synchronization between harmonization of multiple oscillatory components into an integrated signal through phase-coordinated local neuronal spiking^9^, where the phase of one oscillation modulates in harmony with amplitude fluctuations in another oscillation. Widespread slow of coupled chaotic oscillations modulate faster oscillations of local events^10,11^. This is a chaotic system that synchronizes suddenly and completely from one global activity pattern state to another with a minimum duration of transient "phase transition fluctuations". The EEG dynamics show sequences of patterns, each carried by an aperiodic waveform around a chaotic attractor. The synchronization of the EEG network as coupled chaotic oscillators depends on the strength of the coupling, the connectivity topology, and the dynamic characteristics of the system that exists at each brain region. Schwarzian derivatives in high-dimensional chaotic systems exhibit the capacity to create information as well as to destroy it. Chaotic dynamics endows brains with the capacity to create new kinds of trials by which to generalize across inputs from equivalent sensory receptors. Chaos in the model arises when the mesoscopic populations are interconnected with the cortex so that each excites the other^10^ in the continuing update of cortical populations as shown. Each has its characteristic frequency, but they differ and cannot agree. Neither can escape the other so that stable aperiodic oscillation persists as shown in Fig. 1. If the three parts are disconnected, the chaos in the model disappears^12,13^.

We propose a Schwarzian derivative which is invariant under von Bertalanffy’s model^14,15^ of phase growth. This model is a family of unimodal inverse transformation map as which it can be used to determine the EEG dynamics of higher derivatives. With the neuron populations considered in Bertalanffy’s growth model, the size of its activated pool is inversely related to the frequency of synchronization. The slower the oscillation, the more neurons can participate in a larger size pool hence the integrated mean-field is larger. Perturbations at slow frequencies like 4–8 Hz EEG band can trigger a cascade chain of energy dissipations mixing up to higher gamma frequencies (20–50 Hz) which means that widespread slow oscillations modulate faster local EEG oscillations as shown in Fig. 3. We consider the intrinsic growth rate estimation, $r$, that represents a dimensional feedback coupling constant as shown in Figs. 9 and 10. The corresponding brain dynamics undergo stable evolution but for one fixed point solution. The dynamics are in metastability conditions in period doubling and chaotic regions if $\frac{5}{3} < r < \frac{{3^{3} }}{{2^{2} }}$, then the interval $\left[ {f_{r}^{2} \left( c \right),f_{r} \left( c \right)} \right]$ is forward invariant with basin of attraction $\left] {0,1} \right[;$

In terms of entropy change ($\Delta E$) in Schwarzian values of von Bertalanffy functions before medication, this disorder is high in bipolar patients over all electrodes. After medication, those entropy change (∆E) remain low for all electrodes as shown in Figs. 7 and 8.

**Figure 12:**
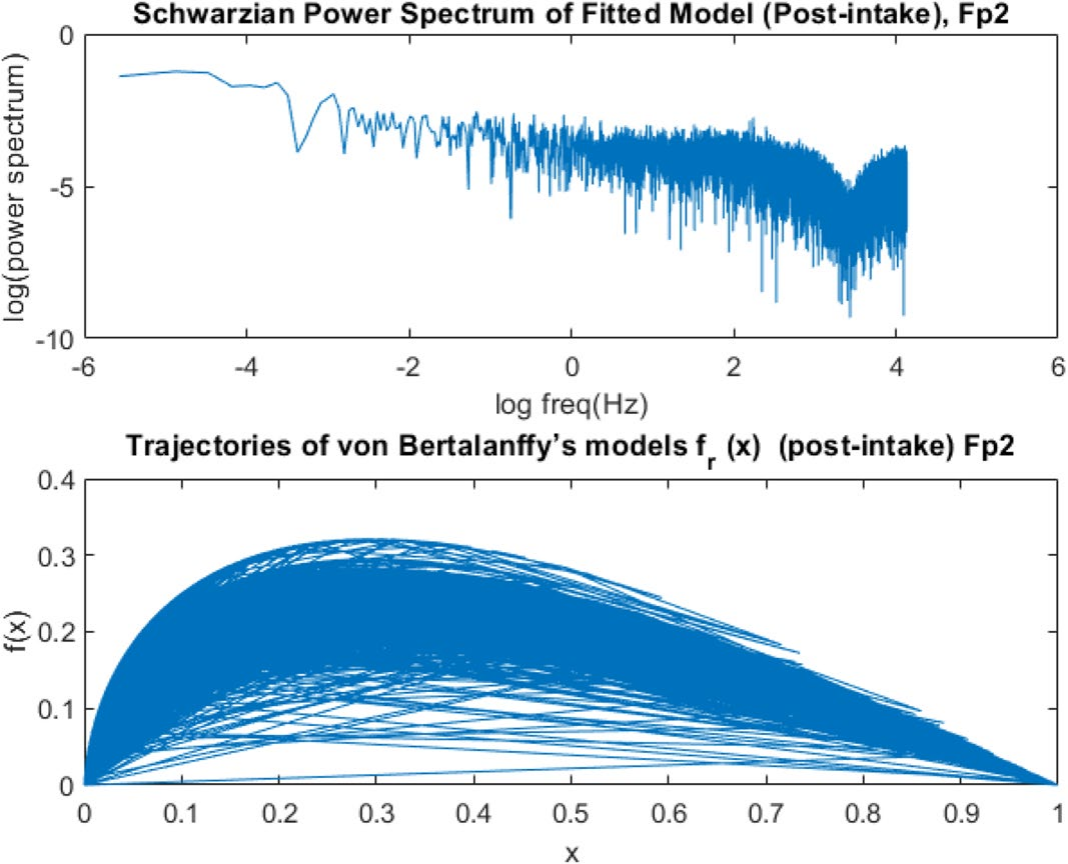
Power Spectrogram of Schwarzian and von Bertalanffy’s EEG Phase Growth Model after medication for Fp2 electrode for an interval of 30,000-time points of T = 500 time points for each segment at fs = 125 Hz.

However, in the classical EEG power spectrum of Schwarzian dynamics, this change could not be observed due to the nonstationary dynamics of EEG signals over different time segments as shown in Figs. 11 and 12. In qEEG theory, the power-density spectrum of EEG is often calculated as the average of many spectra derived on individual time intervals. The determination of chaotic invariants in the frequency domain will not be a solution.

According to our entropy change ($\Delta E$) results, there was no statistical significance in two patients between pre and post-medication after correction. Subject 2 returned from the rope on the verge of suicide. In subject 8, there was a diagnosis of non-Hodgkin lymphoma after this research was over. The entropy change of chaotic transition was first examined in trait bipolar disorder, in other words, bipolar spectrum disorder.

According to our results, lithium carbonate affects the strong chaos spectrum and removes narrow boundaries. We concluded that EEG dynamics covers a wide range of a period doubling and chaotic set of frequencies through the delta, beta, alpha, gamma, etc. bands in a given topology. Intrinsic growth rate estimation, $r$ is a coupling constant is a mathematical method for the precise prediction of chaotic points in the scrambling EEG dynamics. In our discussion, Schwarzian dynamics of von Bertalanffy’s model verifies the dynamics in Period doubling and chaotic regions with a basin of attraction $\left] {0,1} \right[;{ }$ and finally chaotic semistable invariants in [0, 1].

The Lyapunov coefficient is being positive near the origin which shows chaotic semistable invariants where phase growth is in the terms of average $L^{p}$- norms as an estimator of free energy that is approximated by from the square of the EEG phase growth on spheres of radius $r > 0$ as r → ∞ for $1 < p < \infty$ (Figs. 1, 7).

## Data Availability

All data and material archived at our institution according to İnformation and Consent Form on processing and protection of Personal Data.
